# Case report: application of pharmacogenetics in the personalized treatment of an elderly patient with a major depressive episode

**DOI:** 10.3389/fpsyt.2023.1250253

**Published:** 2023-08-07

**Authors:** Milica Pjevac, Sara Redenšek Trampuž, Tanja Blagus, Vita Dolžan, Jurij Bon

**Affiliations:** ^1^Centre for Clinical Psychiatry, University Psychiatric Clinic Ljubljana, Ljubljana, Slovenia; ^2^Pharmacogenetics Laboratory, Institute of Biochemistry and Molecular Genetics, Faculty of Medicine, University of Ljubljana, Ljubljana, Slovenia; ^3^Department of Psychiatry, Faculty of Medicine, University of Ljubljana, Ljubljana, Slovenia

**Keywords:** personalized therapy, pharmacogenetics, cytochrome P450 enzymes, depression, antidepressants, antipsychotics, case report

## Abstract

**Background:**

Pharmacogenetic analyses can predict interpersonal differences in response to psychopharmacotherapy, which greatly facilitates the selection of the most effective medication at optimal doses. By personalizing therapy in this way, we can minimize adverse drug reactions (ADR) and prevent polypharmacy. Most psychotropic medications are metabolized by the cytochrome P450 enzymes CYP2D6, CYP2C19, and CYPA3A4, which influence drug metabolism and concentration, affecting both efficacy and the occurrence of ADR. The relationships between genetic variations and enzymatic activity allow pharmacogenetic analysis to provide important data for optimal drug selection. The following case report illustrates the impact of pharmacogenetic analysis on the course of pharmacologic treatment in an elderly patient with a major depressive episode.

**Methods:**

We present a case of a 79-year-old patient treated for severe depression with psychotic symptoms. We collected data on treatment selection and response to treatment before and after pharmacogenetic analysis. For pharmacogenetic analysis, common functional variants in *CYP1A2*, *CYP3A4*, *CYP2B6*, *CYP2C19*, and *CYP2D6* were genotyped, and corresponding evidence-based treatment recommendations were prepared.

**Results:**

The patient suffered from lack of efficacy and serious ADR of several medications, resulting in worsening depression and treatment resistance over the course of several months of treatment. Pharmacogenetic analysis provided important insights into the patient’s pharmacokinetic phenotype and allowed us to personalize treatment and achieve remission of the depressive episode.

**Conclusion:**

In the case presented, we have shown how consideration of pharmacogenetic characteristics in an individual patient can improve treatment outcome and patient well-being. Knowledge of the patient’s pharmacogenetic characteristics helped us to personalize treatment, resulting in complete remission of psychopathology. Due to the complexity of psychiatric disorders, the efficacy of combinations of different medications, which are often required in individual patients, cannot be clearly explained. Therefore, it is of great importance to conduct further pharmacokinetic and pharmacogenetic studies to better assess gene-drug interactions in psychopharmacotherapy.

## 1. Introduction

Interindividual differences in response to pharmacotherapy play an important part in the treatment of mental disorders. Pharmacotherapy is effective only in a relatively small proportion of patients with mental disorders. In addition to the variable therapeutic effect, ADR of varying intensity often occur. A treatment-resistant form of depression, defined as an inadequate therapeutic response despite adequate treatment with two or more antidepressants, occurs in approximately 30% of patients with depression (1). Similarly, in the treatment of psychotic disorders, only about one-third of patients achieve good long-term remission (2). Additional medications can be used to mitigate the ADR, or if the therapeutic effect is insufficient, combination or augmentation therapy with other medications can be considered. The concomitant use of multiple medications is relatively common, despite the higher risk of ADR. Poor patient adherence to therapy is due, at least in part, to ADR and inadequate therapeutic effect (3). An important determinant of response to therapy is individual pharmacokinetic and pharmacodynamic variability. Common functional variants in genes that code for enzymes, membrane transporters, and therapeutic targets are reflected in interindividual differences in treatment response. Most antidepressants and antipsychotics are metabolized by cytochrome P450 enzymes in the liver, e.g., CYP2D6, CYP2C19, and CYPA3A4. These enzymes were an early focus for clinical use of pharmacogenetics in psychiatry because of the close relationship between genetic variants and enzymatic activity (4). Therefore, pharmacogenetic analysis can provide important data that influence optimal drug selection. Finding the most effective dose with minimal ADR is a critical step toward personalized therapy.

In this case report, we illustrate the importance of considering pharmacogenetic data and recommendations in clinical practice by presenting the case of a patient in whom the application of recommendations successfully changed the course of a previously unsuccessful treatment for a severe depressive episode.

## 2. Case description

A 79-year-old retired teacher was treated for 164 days at the University Psychiatric Clinic Ljubljana (UPCL) for severe depression with psychotic symptoms. She had no previous history of psychiatric treatment. After the death of her husband, she had been living alone for 10 years. She was physically healthy and did not take any medication regularly. There were no particular problems in her family history, and she did not report any substance use disorder.

**Figure 1:**
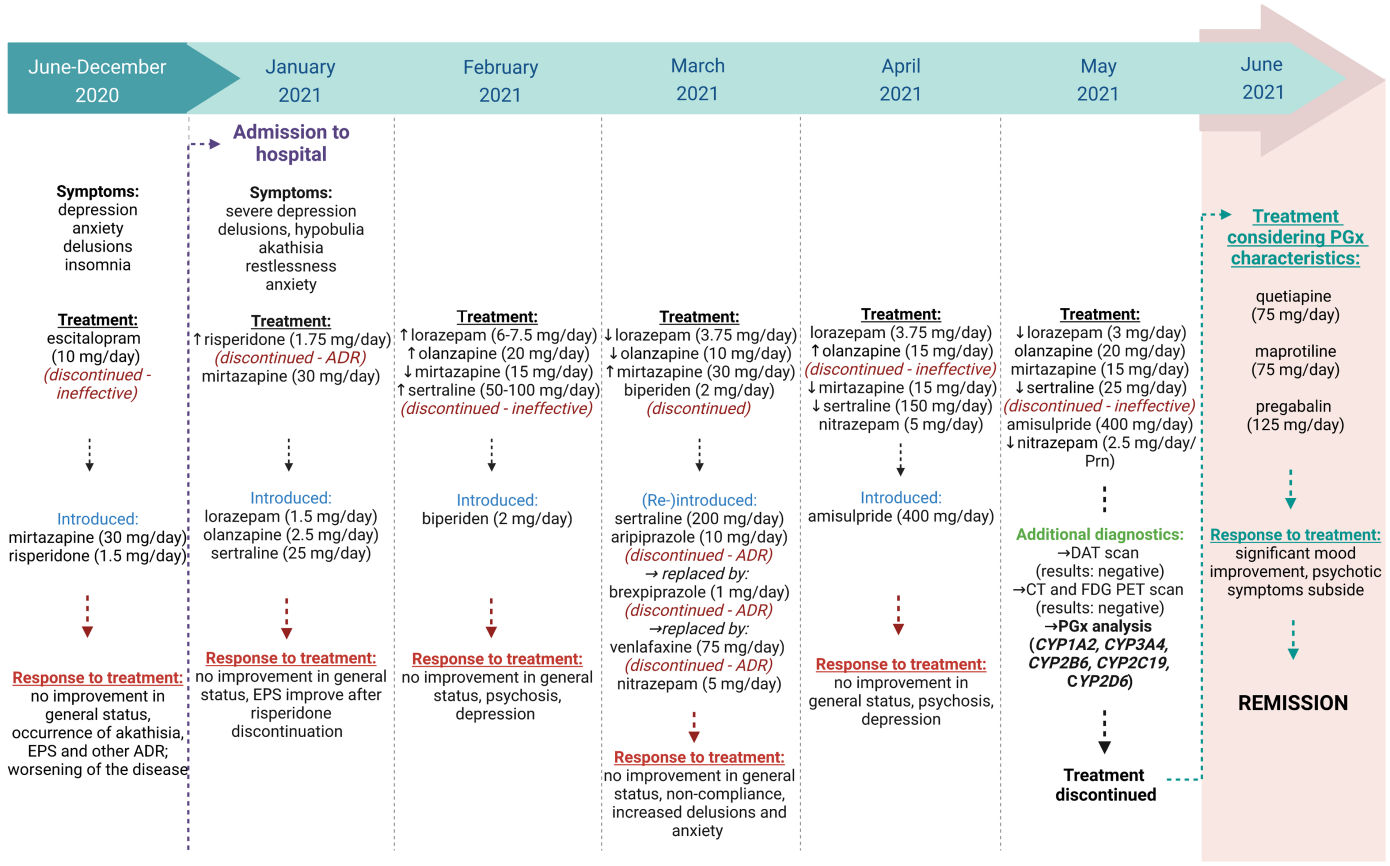
A schematic presentation of the patient’s treatment. Created with Biorender.com.

The patient’s course of treatment is schematically presented in Figure 1.

The first symptoms of depression appeared around 6 months before hospitalization, including anxiety, concerns about her health, lack of will, and anhedonia. Over time, the anxiety and lack of will worsened, her appetite decreased, and she suffered from insomnia. During an outpatient psychiatric evaluation, she was diagnosed with a moderate depressive episode. Initially, she was prescribed escitalopram 10 mg daily, which was switched to mirtazapine due to lack of efficacy. Due to deterioration in daily functioning and self-care, she temporarily moved in with her daughter. After 3 weeks of therapy with 30 mg of mirtazapine daily, improvements in mood, insomnia, and increased energy were noted. However, her concern for her own health and the well-being of her loved ones intensified and gradually took on delusional features. She firmly believed that her digestion had stopped and that something terrible was about to happen to her daughter and grandchildren. In addition to 30 mg of mirtazapine, risperidone was administered at a dose of 0.5 mg three times daily during a follow-up visit. This resulted in increased agitation, despair, a recurrence of decreased willpower, and increased anxiety. The delusional beliefs persisted. Due to the severity of her symptoms, she was admitted to the UPCL.

On admission and for the first few days, she showed marked depressed mood, depressive delusions, delusional interpretations of bodily sensations, and possibly cenesthetic hallucinations. She was, however, fully conscious and oriented, although somewhat suspicious in her interactions. There was no evidence of formal thought disorder, and her cognitive abilities appeared to be intact. She described passive thoughts of death but had no acute suicidal ideation. Initially, therapy was continued with mirtazapine and risperidone. Lorazepam 0.5 mg was additionally prescribed three times daily because of increased anxiety. Motor restlessness was observed and attributed to possible akathisia or depression-related agitation. Other extrapyramidal symptoms (EPS) were also noted: mildly increased muscle tone in the upper limbs and neck, and mild and symmetrical postural tremor. Risperidone was discontinued due to akathisia and EPS. Because of the poor efficacy of mirtazapine monotherapy, combined antidepressant therapy was initiated. A combination of sertraline and olanzapine was introduced. Despite a daily dose of up to 20 mg olanzapine and up to 100 mg sertraline, psychotic symptoms persisted, so aripiprazole was additionally administered. However, as severe akathisia occurred at a dose of 10 mg aripiprazole daily, aripiprazole was replaced by 1 mg brexpiprazole daily. Psychotic symptoms decreased slightly, and she was able to focus on other topics in conversations. At the same time, she continued to receive 75 mg sertraline, 30 mg mirtazapine, 15 mg olanzapine, and 1 mg lorazepam three times daily. Akathisia persisted for several days even at a low dose of 1 mg brexpiprazole daily, but then subsided without a change in medication, resulting in a recurrence of delusions and anxiety. We initiated medication monitoring and found that she had not been taking the prescribed medication, which we attributed to ADR and secondary persecutory delusions. After starting medication monitoring, sertraline 200 mg daily and olanzapine 15 mg daily were confirmed to be ineffective, and brexpiprazole was discontinued because of persistent akathisia. We decided to introduce venlafaxine 75 mg daily. In addition to lorazepam 1 mg three times daily, she also received biperiden 2 mg and olanzapine 20 mg daily. Because of the persistent EPS, a neurologic examination was performed, which revealed mild asymmetric bradykinesia and hypokinesia in the upper limbs (UPDRS stages 1 and 2), Parkinsonian posture, and gait disturbances. Dopamine transporter scintigraphy (DaTSCAN) was performed to confirm presynaptic dopaminergic impairment. The patient had not previously undergone any of the other planned diagnostic tests (CT, EEG) because she had refused them due to her psychotic experiences. Five days after starting venlafaxine 75 mg daily, marked akathisia and reactive worsening of anxiety reappeared. Venlafaxine was therefore discontinued. We temporarily increased lorazepam dose to 1.25 mg in the morning, 2.5 mg in the afternoon, and 2.5 mg in the evening. We also tried amisulpride and gradually increased the dose to 400 mg daily. The dose of sertraline was decreased to 150 mg daily, while mirtazapine was reduced to 15 mg in the evening. Lorazepam was administered regularly, with the dose adjusted to 1 mg three times daily. Nitrazepam 5 mg was introduced for the treatment of insomnia. This also resulted in a slight decrease in anxiety, and akathisia subsided. However, nihilistic and other depressive delusions with bizarre content persisted. With decreased anxiety, a CT brain scan and EEG could be performed, which showed no abnormalities. Laboratory results showed no significant deviations from normal (thyroid hormones, complete blood count, liver function tests, electrolytes, vitamin B12, and folic acid levels). A PET-CT scan with FDG revealed mild diffuse cortical hypometabolism, which was not characteristic of neurodegenerative disease. DaTSCAN excluded parkinsonism due to presynaptic dopaminergic dysfunction. The clinical picture still included depressed mood and depressive delusions. She participated little in ward activities and required considerable assistance with daily activities. In her free time, she tended to withdraw from the company of other patients on the ward.

The examinations performed excluded the differential diagnoses of early-stage dementia or Parkinson’s disease. A clinical psychological examination was performed, but its results were inconclusive because the patient was unable to participate properly. However, based on the low cognitive screening test results (MMSE 25/30, ACE 83/100), some type of dementia syndrome was suspected. The cognitive decline was consistent with the picture of pseudodementia associated with depression.

Due to therapeutic inefficacy of various drug combinations (olanzapine, risperidone, sertraline, mirtazapine, amisulpride) and pronounced ADR with several drugs (aripiprazole, brexpiprazole, risperidone, venlafaxine), a pharmacogenetic analysis was performed.

DNA was isolated from the blood sample collected in EDTA-containing using the E.Z.N.A. SQ Blood Kit II (Omega bio-tek), according to the manufacturer’s instructions. The presence of *CYP2D6* gene deletion (*5) or duplication (*xN) was tested using long-range PCR (Long-Range PCR, Biotechrabbit GmbH) and the amplicons were visualised after agarose gel electrophoresis. The other genetic variants (CYP1A2*1F; CYP3A4*22; CYP2B6*4, *6, *9; CYP2C19*2, *3, *4A/B, *5, *6, *8, *10, *9, *17; and CYP2D6*3, *4, *6, *8, *9, *10, *14A/B, *17, *41) were analysed using commercial KASPar SNP Genotyping Assay kits (LGC Group), which enable the detection of products using fluorescence. All analyses were performed in duplicate in the presence of appropriate negative and positive controls for all genotypes and additional control samples.

## 3. Results and discussion

The patient presented here experienced a plethora of treatment failures, both in terms of inefficacy and ADR (see Figure 1). Based on the list of prescribed drugs and their metabolic pathways, *CYP1A2*, *CYP3A4*, *CYP2B6*, *CYP2C19*, and *CYP2D6* were selected for genotyping for various genetic polymorphisms. Genotyping results and their corresponding phenotypes are shown in Table 1.

**Table 1:** Summary of the genotyping analysis and the patient’s phenotypes.

Gene	Polymorphism	Patient’s genotype	Polymorphic allele contribution	Patient’s phenotype
*CYP1A2*	*1F	*1F/*1F	Increased enzyme activity	UM (DPWG)
*CYP3A4*	*22	*1/*22	Decreased enzyme activity	IM (DPWG)
*CYP2B6*	*4, *6, *9	*1/*4	Increased enzyme activity	RM (CPIC)
*CYP2C19*	*2, *3, *4A/B, *5, *6, *8, *10, *9, *17	*1/*17	Increased enzyme activity	NM (DPWG) RM (CPIC)
*CYP2D6*	*3, *4, *5, *6, *8, *9, *10, *14A/B, *17, *41, xN	*1/*41	Decreased enzyme activity	NM (DPWG, CPIC)

NM, normal metabolizer; IM, intermediate metabolizer; RM, rapid metabolizer; UM, ultra-rapid metabolizer; DPWG, Dutch Pharmacogenomic Working Group; CPIC, The Clinical Pharmacogenetics Implementation Consortium.

Marked in bold are polymorphic alleles that are present in the patient and contribute to changes in the activity of metabolizing enzymes.

Briefly, typical antipsychotics (chlorpromazine, fluphenazine, perphenazine, promethazine, thioridazine), some selective serotonin reuptake inhibitors (SSRIs), and serotonin-norepinephrine reuptake inhibitors (SNRIs) are metabolized predominantly *via* CYP2D6, whereas the metabolism of atypical antipsychotics (aripiprazole, brexpiprazole, iloperidone, risperidone), noradrenergic specific serotonergic antidepressants (NaSSAs), haloperidol, and venlafaxine is also catalyzed by CYP3A4 (5). Clozapine and olanzapine are metabolized predominantly via CYP1A2 and to a lesser extent *via* CYP2D6 and CYP3A4, while most SSRIs are metabolized primarily via CYP2C19 (5–7). Finally, CYP2B6 is an important enzyme for the metabolism of sertraline (8).

The presented patient carries two polymorphic alleles (CYP2D6*41, CYP23A4*22) that contribute to lower activity of metabolizing enzymes, which could lead to impaired conversion of drugs into inactive metabolites, resulting in a higher risk of developing ADR. On the other hand, lower activity could also lead to lower drug efficacy if the parent compound needs to be activated *via* these enzymes. For other CYP enzymes analysed, the patient carries multiple polymorphic alleles (CYP1A2*1F, CYP2C19*17, CYP2B6*4) that result in increased enzyme activity, meaning that the patient could be susceptible to decreased drug efficacy as active compounds are neutralized shortly after drug administration.

Considering the genotypes and phenotypes, many of the treatment-related problems can be explained. Treatment was initiated with the administration of mirtazapine as an atypical tetracyclic antidepressant and risperidone as an atypical antipsychotic. During the course of treatment, mirtazapine proved ineffective, whereas risperidone led to the development of extrapyramidal symptoms (EPS), with akathisia being the most severe. CYP1A2, CYP2D6, and CYP3A4 are responsible for the metabolism of mirtazapine, the latter two being the most important (9, 10). Unfortunately, the lack of efficacy of mirtazapine cannot be explained by the fact that the activity of the crucial metabolizing enzymes was decreased. There is still no strong evidence or pharmacogenetic recommendations for mirtazapine and its gene interactions. Similarly, there are no dose adjustment recommendations for risperidone in CYP2D6*1/*41 (NM). However, decreased CYP2D6 enzyme activity suggests a tendency for drug-gene interactions to cause the occurrence of ADR in risperidone treatment, although no clinically relevant associations have been demonstrated to date (11). Nevertheless, plasma concentrations of 9-hydroxyrisperidone, the equipotent metabolite of risperidone, are known to be decreased in carriers of CYP2D6*41, implying higher plasma concentrations of risperidone (11, 12). Since risperidone is more likely to cross the blood–brain barrier than 9-hydroxyriperidone, this may explain the development of ADR originating from the central nervous system, and thus EPS (13).

When the patient’s treatment was continued, olanzapine also proved ineffective. This may have been because the patient was a CYP1A2*1F/*1F (UM). Carriers of this genotype metabolize olanzapine more rapidly than normal metabolizers, resulting in significantly lower concentrations of the active drug compound in the blood. CYP2D6 and CYP3A4 also contribute to the metabolism of olanzapine, but to a much lesser extent (11).

Sertraline is extensively metabolized in the liver via several different CYP enzymes, CYP2C19 being the most important. However, CYP2B6, CYP2D6, and CYP3A4 also play a role. Slightly lower serum concentrations of sertraline and its active form have been demonstrated in Scandinavian patients with the *CYP2C19* *17 allele (14), suggesting that this phenotype may contribute to lower efficacy of sertraline as observed in the presented patient. Currently, there is insufficient evidence and pharmacogenetic recommendations for the CYP2C19*1/*17 phenotype (NM) based on the Dutch Pharmacogenetic Working Group (DPWG), but there are recommendations based on a combination of genotypes for *CYP2B6* and *CYP2C19* according to the Clinical Pharmacogenetics Implementation Consortium (CPIC). Because the patient carries two alleles (CYP2C19*17 and CYP2B6*4) associated with increased metabolism of sertraline to a less active compound, it is recommended to consider titrating sertraline to a higher maintenance dose or switching to a clinically appropriate alternative antidepressant that is not predominantly metabolized by CYP2C19 or CYP2B6 if a patient does not respond adequately to the recommended initial/maintenance dose of sertraline (15, 16). In addition, lower CYP2D6 and CYP3A4 activity in the presented patient could affect the response to sertraline. However, there is no clear evidence for the clinical relevance of dose adjustments or the occurrence of ADR/efficacy during treatment with sertraline in these phenotypes. Therefore, the patient’s non-response to sertraline treatment is more likely related to increased activity of CYP2C19 and CYP2B6. Knowledge of the patient’s *CYP2C19* genotype/phenotype could also explain the ineffectiveness of escitalopram, the first medication prescribed to the patient when she was diagnosed with a depressive episode. CPIC, but not DPWG, provides the explanation that patients carrying the *17 allele may have lower plasma concentrations of escitalopram, indicating increased metabolism of escitalopram to a less active compound and thus a lower likelihood of clinical benefit. If PGx information were available earlier, the patient’s depression could be successfully treated before hospitalization by increasing the escitalopram dose as recommended by the CPIC (8).

Because the patient did not respond well to the above therapy, the medication was changed to aripiprazole, brexpiprazole, and venlafaxine. After the introduction of the new treatment regimen, several ADR occurred that can be explained, at least in part, by the patient’s pharmacogenetic characteristics. All of these drugs are metabolized mainly via CYP2D6. Since the patient was a carrier of the CYP2D6*41 allele, metabolism *via* this enzyme might be reduced, leading to higher plasma concentrations of the drugs and thus to various ADR (8, 11). Importantly, there are pharmacogenetic recommendations for the *CYP2D6*-aripiprazole gene-drug pair, but only for the “poor metabolizers” (PM). These recommendations state that patients with a *CYP2D6* PM phenotype should not be given more than 10 mg/day or 300 mg/month (68%–75% of the normal maximum dose of aripiprazole) (11). In addition, aripiprazole plasma concentrations may also be elevated due to genetically low CYP3A4 activity, further increasing the risk for the occurrence of ADR (11). Because brexpiprazole is metabolized in a similar manner to aripiprazole, similar consequences in terms of plasma concentrations and possible ADR are expected. Pharmacogenetic recommendations for brexpiprazole state that half the normal dose should be used for *CYP2D6* PMs, which is even less compared to aripiprazole (11). In addition, the patient experienced akathisia while taking venlafaxine. The akathisia could possibly be explained by the fact that the patient was a carrier of CYP2D6*41, meaning that venlafaxine was metabolized to a lesser extent to O-desmethylvenlafaxine. Heterozygous CYP2D6*1/*41 are currently classified as NM, so there are no recommendations for patients with this genotype.

However, regardless of the current phenotype classification, several studies reported that allele*41 decreases the activity of the enzyme to a greater extent as agreed between CPIC and DPWG in 2019 (17–19). Indeed, a consensus has been reached that the NM phenotype is assigned when the activity score is between 1.25 and 2.25, whereas a value below 1.25 indicates a IM phenotype (20). According to this consensus, the contribution of the CYP2D6*41 allele to decreased enzyme activity is estimated to be 0.5, implying that the *1/*41 diplotype has a total score of 1.5 and is therefore NM. Doubts about this consensus on scores were recently raised by the group of Jukić et al. (18), who demonstrated that the activity score for the CYP2D6*41 allele is close to 0.18, especially when risperidone, aripiprazole, and venlafaxine are administered (0.14, 0.26, and 0.21, respectively), and is not equal to 0.5 as assumed by the consensus. Similar results were obtained by Haslemo et al. (19), who found in their study of a large patient population that the score for residual enzyme activity was only 9.5% in the case of venlafaxine administration. Considering these data, the presented patient could be classified as IM rather than NM.

Finally, CYP2D6 activity could also be reduced by sertraline, which is a known weak inhibitor of CYP2D6 (21). With a consequent reduction in CYP2D6 activity, the patient’s CYP2D6 phenotype could shift toward IM or even PM. This could increase the likelihood of ADR due to treatment with risperidone, aripiprazole, brexpiprazole, and venlafaxine, all taken concomitantly with sertraline.

Once the pharmacogenetic results were available, the patient’s genetic characteristics were considered when deciding about the new course of treatment. Because certain drugs were selected for which there were no corresponding pharmacogenetic recommendations, their doses were titrated slowly and carefully. Quetiapine, an atypical antipsychotic, is metabolized mainly via CYP3A4 (22). Because of the patient’s decreased CYP3A4 activity, quetiapine was introduced slowly and under close monitoring. We started with a dose of 12.5 mg in the evening and gradually increased it to 75 mg in the evening. Similarly, maprotiline, a tetracyclic antidepressant, was introduced with caution because of its metabolism via CYP2D6 and CYP1A2 (23). We started with an initial dose of 25 mg and gradually increased it to 75 mg daily. In the titration phase, we focused on the joint effects of maprotiline and quetiapine, particularly with regard to the possible anticholinergic ADR and QTc interval prolongation. For the treatment of anxiety, pregabalin, which is metabolized by the liver to a very low extent (24), was administered at a dose of 25 mg in the morning and at noon and 75 mg in the evening. The patient’s mood improved markedly within 4 weeks, and psychotic symptoms disappeared completely.

## 4. Conclusion

In the case presented, we have shown how consideration of pharmacogenetic characteristics in an individual patient can improve treatment outcome and patient well-being. Treatment without consideration of patients’ pharmacogenetic characteristics resulted in ineffective therapy even at high or maximal doses of antidepressants and antipsychotics. In addition, low doses of some antidepressants and antipsychotics caused the occurrence of serious and complicating ADR. Knowledge of the patient’s pharmacogenetic characteristics helped us to personalize treatment, resulting in complete remission of psychopathology. However, due to the complexity of psychiatric disorders and the combinations of different medications that are often required in individual treatment, response and efficacy cannot be clearly explained. As is often observed in pharmacogenetic studies of cytochrome enzyme gene alleles, some results are consistent with expectations based on previous data from the literature, whereas others aren’t. Therefore, it is of great importance to conduct additional pharmacokinetic and pharmacogenetic studies to better assess gene-drug interactions. This knowledge will help us to adjust dosage and combinations of prescribed drugs to personalize the treatment and increase its safety and efficacy.

## Data availability statement

The data presented in the case report are available upon reasonable request. Further inquiries may be directed to the corresponding authors.

## Ethics statement

Ethical approval was not required for the case report involving humans in accordance with the local legislation and institutional requirements. Written informed consent was obtained from the individual(s) for the publication of any potentially identifiable images or data included in this article.

## Author contributions

MP, JB, and VD contributed to conception and design of the case report. MP organized the data. ST and TB performed the pharmacogenetic analysis. MP, ST, TB, and JB wrote the first draft of the manuscript. TB and ST created the figures. All authors contributed to the article and approved the submitted version.

## Funding

This research was funded by Slovenian research agency (grants P5-0110, P1-0170, J3-4533).

## Conflict of interest

The authors declare that the research was conducted in the absence of any commercial or financial relationships that could be construed as a potential conflict of interest.

## Publisher’s note

All claims expressed in this article are solely those of the authors and do not necessarily represent those of their affiliated organizations, or those of the publisher, the editors and the reviewers. Any product that may be evaluated in this article, or claim that may be made by its manufacturer, is not guaranteed or endorsed by the publisher.

## References

[1] Bahr R Lopez A Rey JA. Intranasal Esketamine (SpravatoTM) for use in treatment-resistant depression in conjunction with an oral antidepressant. P T. (2019) 44:340–75. PMID: 31160868 PMC6534172

[2] Haddad PM Correll CU. The acute efficacy of antipsychotics in schizophrenia: a review of recent meta-analyses. Ther Adv Psychopharmacol. (2018) 8:303–18. doi: 10.1177/2045125318781475, PMID: 30344997 PMC6180374

[3] García S Martínez-Cengotitabengoa M López-Zurbano S Zorrilla I López P Vieta E. Adherence to antipsychotic medication in bipolar disorder and schizophrenic patients: a systematic review. J Clin Psychopharmacol. (2016) 36:355–71. doi: 10.1097/JCP.0000000000000523, PMID: 27307187 PMC4932152

[4] van Schaik RHN Müller DJ Serretti A Ingelman-Sundberg M. Pharmacogenetics in psychiatry: an update on clinical usability. Front Pharmacol. (2020) 11:575540. doi: 10.3389/fphar.2020.575540, PMID: 33041820 PMC7518035

[5] Zanger UM Schwab M. Cytochrome P450 enzymes in drug metabolism: regulation of gene expression, enzyme activities, and impact of genetic variation. Pharmacol Ther. (2013) 138:103–41. doi: 10.1016/j.pharmthera.2012.12.007, PMID: 23333322

[6] Bertilsson L Dahl ML Dalén P Al-Shurbaji A. Molecular genetics of CYP2D6: clinical relevance with focus on psychotropic drugs. Br J Clin Pharmacol. (2002) 53:111–22. doi: 10.1046/j.0306-5251.2001.01548.x, PMID: 11851634 PMC1874287

[7] Ingelman-Sundberg M Sim SC Gomez A Rodriguez-Antona C. Influence of cytochrome P450 polymorphisms on drug therapies: pharmacogenetic, pharmacoepigenetic and clinical aspects. Pharmacol Ther. (2007) 116:496–526. doi: 10.1016/j.pharmthera.2007.09.004, PMID: 18001838

[8] Bousman CA Stevenson JM Ramsey LB Sangkuhl K Hicks JK Strawn JR. Clinical pharmacogenetics implementation consortium (CPIC) guideline for CYP2D6, CYP2C19, CYP2B6, SLC6A4, and HTR2A genotypes and serotonin reuptake inhibitor antidepressants. Clin Pharmacol Ther. (2023) 114:51–68. doi: 10.1002/cpt.2903, PMID: 37032427 PMC10564324

[9] Anttila SA Leinonen EV. A review of the pharmacological and clinical profile of mirtazapine. CNS Drug Rev. (2001) 7:249–64. doi: 10.1111/j.1527-3458.2001.tb00198.x, PMID: 11607047 PMC6494141

[10] Al-Majed A Bakheit AH Alharbi RM Abdel Aziz HA. Mirtazapine. Profiles Drug Subst Excip Relat Methodol. (2018) 43:209–54. doi: 10.1016/bs.podrm.2018.01.002 29678261

[11] Beunk L Nijenhuis M Soree B de Boer-Veger NJ Buunk AM Guchelaar HJ. Dutch Pharmacogenetics Working Group (DPWG) guideline for the gene-drug interaction between CYP2D6, CYP3A4 and CYP1A2 and antipsychotics. Eur J Hum Genet. (2023). doi: 10.1038/s41431-023-01347-3 [Epub ahead of print], PMID: 37002327 PMC10923774

[12] Jukic MM Smith RL Haslemo T Molden E Ingelman-Sundberg M. Effect of CYP2D6 genotype on exposure and efficacy of risperidone and aripiprazole: a retrospective, cohort study. Lancet Psychiatry. (2019) 6:418–26. doi: 10.1016/S2215-0366(19)30088-4, PMID: 31000417

[13] Calarge CA Miller DD. Predictors of risperidone and 9-hydroxyrisperidone serum concentration in children and adolescents. J Child Adolesc Psychopharmacol. (2011) 21:163–9. doi: 10.1089/cap.2010.0038, PMID: 21486167 PMC3080754

[14] Bråten LS Haslemo T Jukic MM Ingelman-Sundberg M Molden E Kringen MK. Impact of CYP2C19 genotype on sertraline exposure in 1200 Scandinavian patients. Neuropsychopharmacology. (2020) 45:570–6. doi: 10.1038/s41386-019-0554-x, PMID: 31649299 PMC6969041

[15] Whirl-Carrillo M Huddart R Gong L Sangkuhl K Thorn CF Whaley R. An evidence-based framework for evaluating pharmacogenomics knowledge for personalized medicine. Clin Pharmacol Ther. (2021) 110:563–72. doi: 10.1002/cpt.2350, PMID: 34216021 PMC8457105

[16] Whirl-Carrillo M McDonagh EM Hebert JM Gong L Sangkuhl K Thorn CF. Pharmacogenomics knowledge for personalized medicine. Clin Pharmacol Ther. (2012) 92:414–7. doi: 10.1038/clpt.2012.96, PMID: 22992668 PMC3660037

[17] Molden E Jukić MM. CYP2D6 reduced function variants and genotype/phenotype translations of CYP2D6 intermediate metabolizers: implications for personalized drug dosing in psychiatry. Front Pharmacol. (2021) 12:650750. doi: 10.3389/fphar.2021.650750 33967790 PMC8100508

[18] Jukić MM Smith RL Molden E Ingelman-Sundberg M. Evaluation of the CYP2D6 haplotype activity scores based on metabolic ratios of 4,700 patients treated with three different CYP2D6 substrates. Clin Pharmacol Ther. (2021) 110:750–8. doi: 10.1002/cpt.2246, PMID: 33792048

[19] Haslemo T Eliasson E Jukić MM Ingelman-Sundberg M Molden E. Significantly lower CYP2D6 metabolism measured as the O/N-desmethylvenlafaxine metabolic ratio in carriers of CYP2D6*41 versus CYP2D6*9 or CYP2D6*10: a study on therapeutic drug monitoring data from 1003 genotyped Scandinavian patients. Br J Clin Pharmacol. (2019) 85:194–201. doi: 10.1111/bcp.13788 30312494 PMC6303206

[20] Caudle KE Sangkuhl K Whirl-Carrillo M Swen JJ Haidar CE Klein TE. Standardizing CYP2D6 genotype to phenotype translation: consensus recommendations from the clinical pharmacogenetics implementation consortium and Dutch pharmacogenetics working group. Clin Transl Sci. (2020) 13:116–24. doi: 10.1111/cts.12692 31647186 PMC6951851

[21] Sproule BA Otton SV Cheung SW Zhong XH Romach MK Sellers EM. CYP2D6 inhibition in patients treated with sertraline. J Clin Psychopharmacol. (1997) 17:102–6. doi: 10.1097/00004714-199704000-00007, PMID: 10950472

[22] Spina E de Leon J. Metabolic drug interactions with newer antipsychotics: a comparative review. Basic Clin Pharmacol Toxicol. (2007) 100:4–22. doi: 10.1111/j.1742-7843.2007.00017.x, PMID: 17214606

[23] Brachtendorf L Jetter A Beckurts KT Hölscher AH Fuhr U. Cytochrome P450 enzymes contributing to demethylation of maprotiline in man. Pharmacol Toxicol. (2002) 90:144–9. doi: 10.1034/j.1600-0773.2002.900306.x, PMID: 12071336

[24] Ben-Menachem E. Pregabalin pharmacology and its relevance to clinical practice. Epilepsia. (2004) 45:13–8. doi: 10.1111/j.0013-9580.2004.455003.x 15315511

